# Application of Electrospinning in Antibacterial Field

**DOI:** 10.3390/nano11071822

**Published:** 2021-07-14

**Authors:** Honghai Li, Xin Chen, Weipeng Lu, Jie Wang, Yisheng Xu, Yanchuan Guo

**Affiliations:** 1 Key Laboratory of Photochemical Conversion and Optoelectronic Material, Technical Institute of Physics and Chemistry, Chinese Academy of Sciences, Beijing 100190, China; 15061881525@163.com (H.L.); chenxin@mail.ipc.ac.cn (X.C.); 2 School of Chemical Engineering, East China University of Science and Technology, Shanghai 200237, China; 3 School of Future Technology, University of Chinese Academy of Sciences, Beijing 100049, China

**Keywords:** electrospinning, antibacterial nanofibers, structure, application

## Abstract

In recent years, electrospun nanofibers have attracted extensive attention due to their large specific surface area, high porosity, and controllable shape. Among the many applications of electrospinning, electrospun nanofibers used in fields such as tissue engineering, food packaging, and air purification often require some antibacterial properties. This paper expounds the development potential of electrospinning in the antibacterial field from four aspects: fiber morphology, antibacterial materials, antibacterial mechanism, and application fields. The effects of fiber morphology and antibacterial materials on the antibacterial activity and characteristics are first presented, then followed by a discussion of the antibacterial mechanisms and influencing factors of these materials. Typical application examples of antibacterial nanofibers are presented, which show the good prospects of electrospinning in the antibacterial field.

## 1. Introduction 

Antibacterial materials are important for promoting human health. Among the many preparation technologies of antibacterial materials, nanofibers prepared via electrospinning have the unique advantages of large specific surface area, appropriate porosity, and uniform fiber diameter, which play an important role [1,2,3]. The history of electrospinning originated in the 1930s [4]. It was found that this technology was able to stretch in a polymer solution or melt in an electrostatic field and rapidly obtain nanofibers by evaporating solvent. In 1993, Jayesh Doshi and Darrell H. Reneker systematically summarized electrospinning from the aspects of mechanism and application, and indicated that some of the organic polymers can be electrospun into nanofibers [5]. Since then, this technology has been widely researched [6]. With the rapid development of electrospinning in different applications, a large number of studies show that electrospun nanofibers can have a good antibacterial effect [7,8]. For example, when used in the field of air purification or water treatment, the high specific surface area and porosity of electrospun fiber can make the antibacterial agent fully contact with the medium and play an antibacterial role without affecting the membrane flux [9,10]. In the field of wound dressings or tissue engineering, nanofibers with an extracellular matrix-like structure can play a good antibacterial role without influencing the growth and differentiation of cells [11]. These advantages attracted people in different fields due to their potential applications for antibacterial aspects. Recently, different fiber structures, different antibacterial materials, and different application fields have gradually been developed [12,13,14,15].

However, despite the increasing number of studies, preparation of antibacterial nanofibers via electrospinning has not been systematically summarized. We hope that we can provide some references for scholars who consider matters like which type of nanofiber to prepare. Therefore, for this article we analyzed the antibacterial characteristics of different fiber structures; divided antibacterial materials into synthetic organics, inorganic particles, and natural antibacterial materials; discussed their antibacterial mechanisms; and summarized the application fields of the antibacterial fibers. 

## 2. Structure of Nanofibers

**Figure 1:**
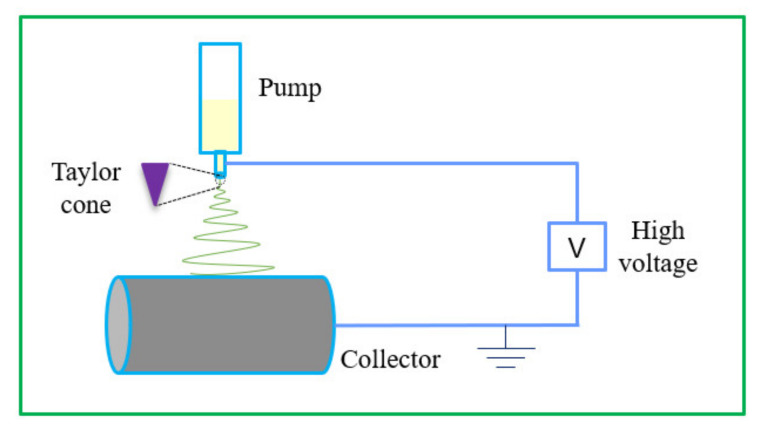
Schematic diagram of a typical electrospinning device.

The process of electrospinning fiber can be divided into three parts. 1. Droplets gather charge and form a Taylor cone in an electrostatic field. 2. When the electric field is strong enough, the droplets eject from the Taylor cone and rotate under the action of a high-voltage electrostatic field. During this process, the jet is elongated rapidly, and the solvent volatilizes rapidly. 3. The jet solidifies on the collector in a random form to form nano-sized fibers [16,17]. In short, the electrospinning device is mainly composed of three parts: a high-voltage power supply, spinneret, and receiver. A typical electrospinning device is shown in Figure 1.

Electrospinning parameters have some common effects on the morphology of fibers. Generally speaking, no matter what electrospinning technology is adopted, parameters will have the same effect on the fiber morphology. The influencing factors can be divided into three categories: solution properties, environmental parameters, and operation parameters. The properties of the solution include volatility, conductivity, concentration, and viscosity. The operating parameters include the distance from the needle to the receiver, the applied voltage, and the flow rate of the solution. The environmental parameters include temperature and humidity. These parameters will affect the shape, size, and consistency of the fiber [18,19,20]. Any parameter exceeding the critical range will lead to spinning failure. For example, polymer solution can only form droplets when the polymer solution is separated by a solvent in a certain dilution state, that is, electrospray. In the semidiluted state, the polymer chains can overlap each other, but this is not enough to form a significant entanglement, and the droplet morphology is still dominant. When the entanglement concentration is reached, a high number of beaded fibers can be obtained. Only when the entanglement concentration is higher than 2–2.5 times the entanglement concentration, the stable and coherent fiber 30 can be obtained [21]. In addition, the volatility of the solvent also affects the morphology and structure of the fiber, such as the size of the pores in the process of forming the porous structure. But when the speed of solvent volatilization is too fast, the nozzle is easy to block, and when the speed of solvent volatilization is too slow, the prepared fibers tend to adhere [19].

Fiber structure has a great impact on antibacterial activity. For example, traditional electrospun fibers mixed with inorganic nanoparticles can continue to be resistant to bacteria, but they cannot express higher antibacterial activity because some nanoparticles are encapsulated in the fibers. The main advantage of core–shell nanofibers is that they can allow for the layered release of drugs in the fibers and be designed with an appropriate drug-release curve according to needs [22]. The antibacterial characteristics of nanofibers with different structures are discussed below. 

### 2.1. Homogeneous Nanofibers

Nanofibers with uniform internal and external composition and smooth and continuous surface can be obtained by electrospinning a homogeneous polymer solution. At present, the main electrospinning devices can be divided into needle electrospinning and needleless electrospinning. In the process of needle electrospinning, droplets first form a Taylor cone at the needle tip. When the electric field is strong enough, the droplets eject from the Taylor cone and eject to the collector, during which the jet is elongated and the solvent volatilizes rapidly. Finally, the jet is solidified on the collector randomly to form nanofibers. In the process of needleless electrospinning, the liquid must flow from the container to the edge of the plate or roller so as to produce multiple jet streams under the action of the electric field [23]. Both methods can prepare homogeneous antibacterial nanofibers with good antibacterial activity by simply mixing an antibacterial composition with the electrospinning solution [24,25,26]. However, in this structure some antibacterial compositions are encapsulated in the nanofibers and the antibacterial activities cannot be engaged at first. Therefore, porous nanofibers with higher specific surface area and the nanofibers with antibacterial components attached to the surface have been developed. In addition, sometimes it is not necessary for nanofibers to engage in high antibacterial activity at the first time, so people have designed core–shell structure nanofibers [27,28].

### 2.2. Nanofibers Mixed with Nanoparticles

From the perspective of fiber structure, nanoparticles can be combined with nanofibers via mixing or surface adhesion. Electrospun fibers mixed with nanoparticles can be obtained by dispersing nanoparticles in a polymer solution or post-treatment of prepared nanofibers. When nanoparticles are dispersed in a polymer solution, the fiber morphology is affected by nanoparticles dispersion, which relates to their size, type, and properties [29,30]. If the nanoparticles agglomerate in the solution, the nanoparticles will be distributed unevenly in the fibers, and even the electrospinning process becomes unstable (such as from blocking needle) [31]. In order to ensure that nanoparticles are evenly dispersed in fibers, the stable dispersion of particles in polymer solutions must be ensured. At present, the nanoparticles in electrospinning mainly include metals, metal oxides, and carbon materials, and their morphology also ranges from a zero-dimensional structure such as Au and Ag nanoparticles [32], one-dimensional Ag nanowires [33], carbon nanotubes [34], two-dimensional clay sheets [35], graphene-based nanosheets, etc. [36,37].

**Figure 2:**
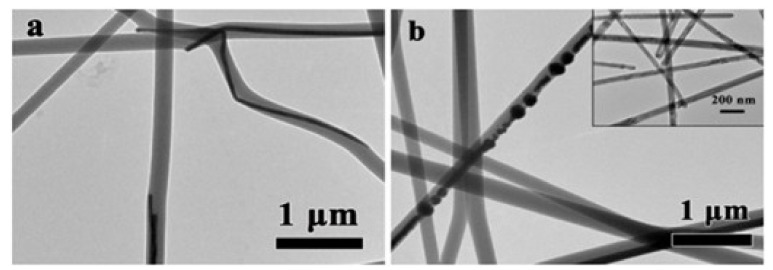
(**a**,**b**) TEM images of Ag NWs/PVA nanofibers with 75 μL Ag nanowire addition, the inset in (**b**) is a TEM image of Ag NWs in a PVA pre-electrospinning solution. Reprinted with permission from ref. [33]. Copyright 2017 Elsevier.

For example, Ag nanowire/polyvinyl alcohol (PVA) composite nanofibers are prepared via single needle electrospinning after adding Ag nanowires into a PVA solution directly [33]. Although some of the Ag nanowires are broken in PVA nanofibers, this does not affect the antibacterial activity of the nanofibers (Figure 2). Besides, Wang mixed graphene oxide (GO) into silk fibroin nanofibers [38] and Bakhsheshi-Rad incorporated GO and silver nanoparticles into poly-L-lactic acid (PLGA) nanofibers, and both of the results showed that the nanofibers had good antibacterial activity and biocompatibility [39].

### 2.3. Nanofibers with Nanoparticles Attached to The Surface 

**Figure 3:**
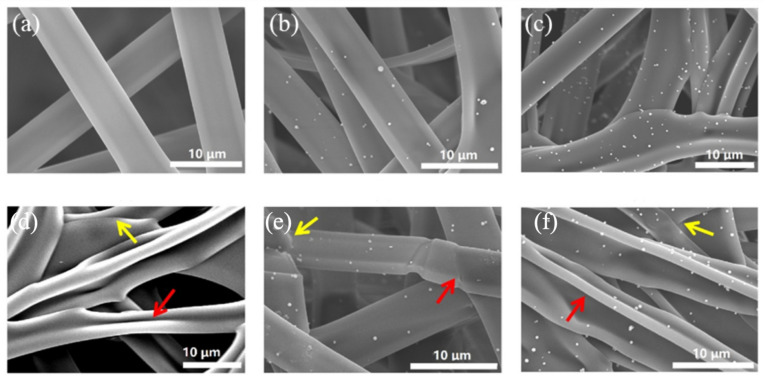
SEM images of (**a**) GZ0′, (**b**) GZ1′, (**c**) GZ2′, (**d**) GZ0, (**e**) GZ1, and (**f**) GZ2. GZ0′, GZ1′, and GZ2′ represent gelatin/ZnO fibers with a concentration of 0%, 0.1%, and 0.25% in the ZnO particle solution during the electrospinning process, respectively. GZ0, GZ1, and GZ2 represent GZ0′, GZ1′, and GZ2′ after cross-linking. Referred from [40].

The effect of nanoparticles can be exerted better by attaching nanoparticles to the surface of nanofibers. At present, the most common method to make nanoparticles adhere to the fiber surface is the combination of electrospinning and electrospray, that is, one needle is used to spray a homogeneous spinning solution, and another needle is added to spray the dispersion of nanoparticles. To better exert the antibacterial effect of ZnO nanoparticles, gelatin nanofibers with ZnO attached to the surface were prepared by electrospinning a gelatin solution and an ethanol solution containing ZnO nanoparticles, respectively [40]. SEM images showed that the pure gelatin fibers were uniform and smooth, with ZnO nanoparticles dispersed on the surface of the fibers evenly after adding the nanoparticles into the solution (Figure 3). Cross-sections of the fibers were also observed, and no nanoparticles were found in the fibers. 

Some scholars compared the antibacterial activities of the nanofibers prepared via two methods. Rodríguez-Tobías applied poly(3-hydroxybutyrate) (PHB) to prepare nanofibers using the mixing method and the electrospinning-electrospray method, respectively [41]. The same concentration of ZnO nanoparticles was used in both the hybrid electrospinning and electrospinning/electrospray methods, the antibacterial efficiency value of the former being equal to 3.20 ± 0.15, but that of the latter was only 1.20–1.40 (antibacterial efficiency value should be higher than 2, i.e., able to kill 99.99% of bacteria). Although in this work nanofibers prepared using the electrospinning-electrospray method did not have superior antibacterial properties as expected, and the author’s other characterizations indicated possible causes. For example, TGA showed the nanofibers prepared using the electrospinning-electrospray method had fewer ZnO nanoparticles attached. EDX showed that the aggregation of ZnO and some nanoparticles fell off, which may be the reason for the limited antibacterial activity of PHB/ZnO nanofibers prepared via electrospinning-electrospray.

In addition to combining electrospinning and electrospray, Ranjith deposited ZnO nanoparticles onto the electrospun fibers via atomic layer deposition and then put them into an aqueous solution containing zinc nitrate and hexamethylene tetramine to make the ZnO continue to grow at a low temperature [42]. Zhao decorated the surface of TiO_2_ nanofibers with Ag by electroless plating. Although these scholars did not test the antibacterial properties of the composite nanofibers, the nanoparticles they used are typical antibacterial materials, which can provide ideas for scholars in this field [43].

### 2.4. Nanofibers with Core–Shell Structure

In conventional electrospinning, the active agents are usually simply mixed with a polymer solution to prepare composite fibers. Although active agents can play a role in nanofibers, the explosive release of drugs often occurs, which is not expected in some cases. Emulsion and coaxial electrospinning are two kinds of technologies often used in drug delivery applications as they can improve the burst of drug release by preparing core–shell nanofibers [44,45]. In the nanofibers with this structure, agents can be embedded into core–shell nanofibers and then the polymer fibers in the shell can act as additional barriers to control the drug-release curve.

**Figure 4:**
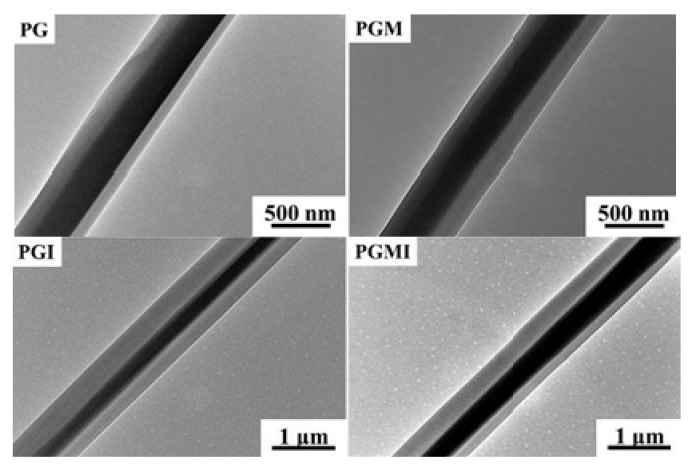
TEM images show the core-sheath structure of coaxial electrospun fibers with different components when electrospun for 30 min. Reprinted with permission from ref. [50]. Copyright 2019 Elsevier.

The needle of the coaxial electrospinning device is composed of an inner needle and an outer needle. When electrospinning, the core fluid and shell fluid meet at the exit of the coaxial needle, and the shell fluid envelops the core fluid to form the jet under the action of the electric field [19]. As solvents, they need to be ensured that they do not mix rapidly when forming Taylor cones to avoid the formation of nanofibers with mixed components. In addition, it also needs to be ensured that the viscosity of the shell solution and the flow rate during spinning are higher than that of the core solution so that the core fiber can be completely wrapped by the shell fiber, which is verified in many coaxial electrospinning literatures [46,47,48,49]. In order to emphasize the synergy of antibacterial and osteogenesis in the bone regeneration process, Gong et al. prepared core–shell nanofibers via coaxial electrospinning, as shown in Figure 4. [50]. Based on the different degradation rates of polycaprolactone (PCL) and gelatin in the core–shell structure, the layered release of moxifloxacin hydrochloride (MOX) and icariin (ICA) was realized, and the core–shell fibers loaded with dual drugs significantly enhanced the osteogenic differentiation of MC3T3-E1 cells. 

The key to forming core–shell nanofibers via emulsion electrospinning is to ensure the phase separation of the two components in the emulsion. In the electrospinning of emulsion, whether it is oil-in-water or water-in-oil type emulsion, the process of jet production will cause emulsion separation due to the solvent evaporation process, resulting in phase separation [51]. For example, Ma et al. dissolved chitosan (CS) and PCL in formic acid (FA) and dichloromethane (DCM), respectively, and then mixed the two solutions in proportions for single-needle electrospinning [52]. The PCL/CS nanofibers exhibited a core–shell structure, where PCL formed a shell as a continuous phase, and CS formed a core as a dispersed phase. 

By selectively removing the core layer of core–shell nanofibers, hollow nanofibers with controllable wall thicknesses can be obtained [53,54]. This structure can also achieve the controlled release of drugs.

### 2.5. Nanofibers with Porous Structure 

Introducing a porous structure to nanofibers can significantly increase the specific surface area of the fibers. There are two main methods to generate pores on nanofibers: (1) phase separation during electrospinning; (2) selective removal of sacrificial phase via post-treatment such as leaching or calcination [55].

Generally speaking, phase separation means that the polymer solution is in a thermodynamically unstable state due to certain reasons, forming polymer-enriched regions with more polymers and polymer-depleted regions with less polymers. During the evaporation of the electrospinning solution, the polymer-enriched regions are solidified into fibers, while the polymer-depleted regions form pore structures [56,57,58]. The phase separation can be divided into three ways: (1) thermally induced phase separation; (2) gas-phase-induced phase separation; (3) non-solvent-induced phase separation [59,60,61]. Chen et al., analyzed the formation process of porous fibers and divided the process into four stages [62]. In the first stage, a uniform solution was ejected from the electrospinning needle. In the second stage, the highly volatile solvent DCM evaporated rapidly, resulting in the decrease of fiber surface temperature. The third stage was divided into two aspects. On the one hand, the phase separation caused by lower temperature led to the polymer-rich and polymer-poor phases on the fibers. On the other hand, water vapor condensed on the surface of the fiber due to the decrease in temperature. In the final stage, the polymer-rich phase formed elliptical nanopores during the drying and solidification process, and the evaporated water droplets formed larger holes on the fiber surface. The post-treatment process was the process of leaching or calcining the fibers, which selectively removed the sacrificial phase from the nanofibers and formed porous structures. The sacrificial phase can be inorganic salt, polymers, block copolymers, etc [63].

**Figure 5:**
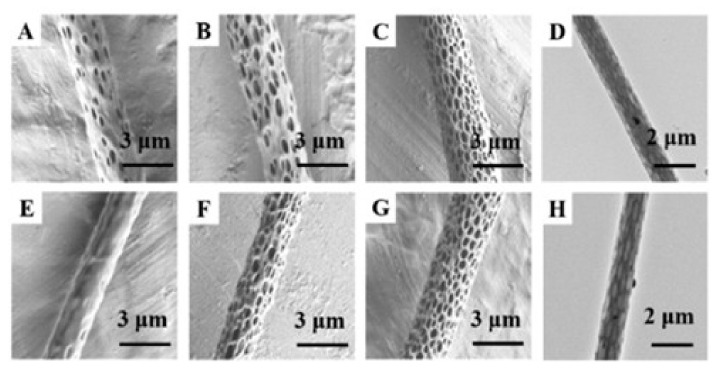
SEM images of porous PLA nanofibers with (DCM/EtOH) (**A**) 90:10 (V/V), (**B**) 95:5 (V/V), and (**C**) 100:0 (V/V). Porous PLA nanofibers were electrospun at (**E**) 20% RH, (**F**) 50% RH, (**G**) 80% RH. TEM images: (**D**) and (**H**). Reprinted with permission from ref. [64]. Copyright 2021 Elsevier.

In order to illustrate the effect of porosity on release rate, Min et al. prepared porous fibers with different pore sizes and pore depths by changing solvents and adjusting the environmental humidity and tested the release of thymus essential oil from fibrous membranes, as shown in Figure 5 [64]. After 5 days, the cumulative release of TEO released from the PLA/TEO/PVA/PEG composite membranes reached 99.87 ± 1.00% (80% RH), 68.16 ± 2.16% (50% RH), and 36.05 ± 1.83% (20%RH), respectively, which proved the promotion of drug release as it relates to the fiber holes.

The porous nanofibers had a higher specific surface area than conventional nanofibers and broadened the application of fiber materials in more fields, such as oil adsorbent, sweat absorbing, and moisture removing materials [65,66].

In general, nanofibers of different structures have different characteristics. Choosing the appropriate structure of nanofibers will help the materials to play a better role and meet the various application needs of researchers.

## 3. Antibacterial Materials

In addition to morphology, materials are also an important factor affecting the antibacterial activity of nanofibers. Electrospinning materials can be divided into two categories: fiber forming materials such as PLA, PVA, cellulose, etc., and antibacterial materials such as silver, quaternary ammonium salt, chitosan, etc. It has been found that most fiber-forming materials have no antibacterial effect. In this chapter, the classification of the materials with antibacterial effects in electrospinning will be described.

### 3.1. Synthetic Organics 

Antibacterial synthetic organics in the field of electrospinning are mainly comprised of various drugs. Electrospun fibers have the characteristics of being nanometer in size and high in porosity, and combined with having a structure similar to a natural extracellular matrix (ECM), electrospun fibers can provide sufficient gas exchange and promote cell adhesion, growth, proliferation, and differentiation on the fibers. These characteristics make them extremely suitable for tissue engineering or drug delivery systems. When the fibers have the required functional properties, such as mechanical strength, degradability, biocompatibility, and antibacterial activities, the fibers can replace the conventional membrane materials to avoid membrane removal surgery and prevent secondary infection [67,68]. Some applications of organic synthetic antibacterial agents in electrospinning are shown in Table 1.

**Figure 6:**
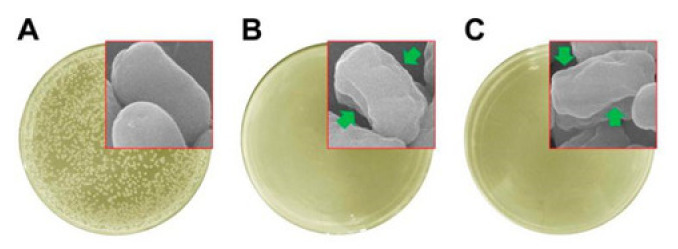
Photographs for the bacterial culture plates of *E. coli* upon a 120 min exposure of (**A**) the control, (**B**) PMMA–DCDMH, and (**C**) PMMA–DBDMH. Reprinted with permission from ref. [78]. Copyright 2016 ACS.

Basically, the combination of antibacterial drugs and cell-promoting drugs can meet people’s various application needs. As shown by Qian, the three-layered fiber membranes prepared using this method are applicable in the field of bone regeneration due to the addition of β-tricalcium phosphate and chlorhexidine (CHX) in the innermost fibers and the outermost fibers, respectively [77]. Bai et al. applied the method of mixing N-halamine with a spinning solution to prepare nanofibers [78]. As shown in Figure 6, the surface of the *E. coli* treated with the fibers collapsed and wrinkled, indicating that the rod-shaped structure had been destroyed and its integrity could not be maintained. In the study of Shi et al., tetracycline hydrochloride (TCH) was first encapsulated by halloysite nanotubes and then mixed into a PLAG solution for electrospinning. The results showed that this dual-container drug delivery system was beneficial to reduce drug burst release and improve the drug release curve. In another study by this author, electrospinning of amoxicillin (AMX) encapsulated by kaolin nanotubes also proved the sustained release of the drug [68]. Besides, the fibers also showed effective antibacterial activity and good cell compatibility. These two studies allow researchers to hypothesize that other clay materials that encapsulate drugs can also be embedded in polymer fibers to improve drug release curves in various biomedical applications.

### 3.2. Inorganic Particles

**Figure 7:**
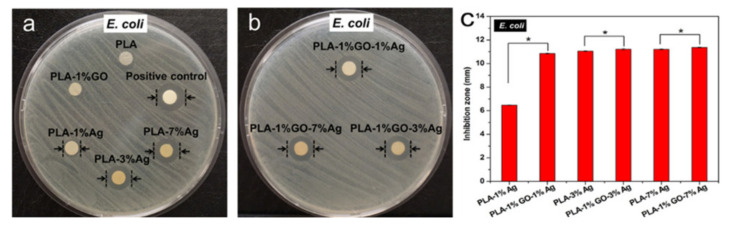
Photographs of agar plates cultivated with *E. coli* and treated with (**a**) PLA, PLA-1%GO, PLA-1%Ag, PLA-3%Ag, PLA-7%Ag, and positive control samples, and (**b**) PLA-1%GO-1%Ag, PLA-1%GO-3%Ag, PLA-1%GO-7%Ag hybrid fibrous mats. (**c**) Antibacterial inhibition zone values determined from (**a**) and (**b**) for the PLA-Ag and PLA-1%GO-Ag nanocomposite fibrous materials. Reprinted with permission from ref. [85]. Copyright 2017 ACS.

Inorganic antibacterial materials tend to be stable and persistent, which gives them a wide range of application prospects. When the size of particles is reduced to the nanoscale, the higher specific surface area and volume ratio makes these particles have different physical and chemical properties compared to ordinary materials. Electrospinning fibers can easily immobilize inorganic nanoparticles, thus providing unique catalytic, optical, and antibacterial properties. Therefore, the inorganic antibacterial materials currently used in electrospinning are mainly nanoscale metals, metal oxides, and carbon materials. Ag is the main metal with antibacterial properties [79]. Metal oxides include TiO_2_, ZnO, MgO, CaO, Al_2_O_3_, Ag_2_O, and CeO_2_ [80,81,82,83,84]. Antibacterial carbon materials are mainly graphene derivatives and carbon nanotubes [34,85,86]. Figure 7 shows agar plates cultivated with *E. coli* and treated with PLA-GO. In the following, representative antibacterial materials in each category are described.

#### 3.2.1. Metal

Ag nanoparticles are a widely studied antibacterial material. They have been introduced into electrospinning of nanofibers for wound dressing, water filtration, and other fields. The Ag/polymer composite nanofibers have good antibacterial activity against a variety of microorganisms. Zhang et al. added prepared Ag nanoparticles into a PVP solution. After obtaining the best electrospinning conditions through orthogonal experiments, the antibacterial properties of the nanofibers with different Ag nanoparticle additions to *E. coli* and *S. aureus* were tested via the absorption method and turbidity method. The results showed that with the increase of Ag nanoparticles, the antibacterial activity of the composite nanofibers against both bacteria was improved [87].

#### 3.2.2. Metal Oxide

ZnO is an important metal oxide material, which is widely used as semiconductor, optical device, piezoelectric device, surface acoustic wave device, sensor, transparent electrode, solar cell, and antibacterial material [88,89]. In addition, ZnO has been classified as GRAS by the FDA, and has shown broad-spectrum antibacterial activity against gram-positive bacteria, gram-negative bacteria, fungi, protozoa, and viruses, which proves its good application prospects in biological and medical fields [90].

Many works have combined ZnO with electrospinning technology to prepare antibacterial nanofibers. Chhabra et al. prepared gelatin nanofiber scaffolds doped with ZnO nanoparticles via electrospinning [31]. The fibers showed no significant antibacterial activity but exhibited better cell proliferation compared to the substrate, which may be attributed to the combination effect of ZnO and endothelial progenitor cells. Figueroa-Lopez et al. prepared nanofibers with oregano essential oil (OEO), ZnO nanoparticles, and a mixture of the two [91]. The results showed that ZnO promoted the antibacterial activity of fibers for a long time. Moreover, compared with PHBV fibers without OEO and ZnO nanoparticles, the hybrid fibers showed less agglomeration of nanoparticles, indicating that the presence of the essential oil was conducive to the dispersion of nanoparticles.

#### 3.2.3. Carbon-Based Nanomaterials

The antibacterial properties of carbon-based nanomaterials have been verified by many studies. They can be divided into zero-dimensional fullerene, carbon dots, graphene quantum dots, one-dimensional carbon nanotubes, two-dimensional graphene, and their derivatives, but currently used in the field of electrospinning antibacterial materials are mainly carbon nanotubes and graphene derivatives [92,93,94]. In the original state, the antibacterial ability of most carbon-based nanomaterials is limited. but their antibacterial activity can be greatly improved via surface functionalization. In addition, carbon-based nanomaterials can be functionalized with specific biologically active molecules and functional groups to improve their targeting ability [95].

Increasing the amount of these carbon-based nanomaterials can effectively enhance the antibacterial activity of the nanofibers without changing their morphology [34,96]. At the same time, the antibacterial activity of carbon nanomaterials can be further improved by introducing heteroatoms or metal/metal oxide nanoparticles [97]. However, there are some differences when introducing metal and metal oxides. When introducing metal nanomaterials, carbon nanomaterials such as GO usually play a role in supporting the growth of Ag nanoparticles to improve the dispersion of nanoparticles and use their own edge cutting way to engage antibacterial activity [98]. When introducing ZnO, TiO_2_, and other metal oxides, in addition to the above effects, the photogenerated electrons generated by the semiconductor may be further transferred to the carbon nanomaterial substrate, slowing down the recombination efficiency of electron hole pairs. This significantly increases the formation of hydroxyl radicals and produces more effective sterilization activity against bacteria [97,99,100]. In addition, when scholars used ionic liquid modification or metal ion coordination methods to pre-treat the carbon-based nanomaterials that electrospun into the nanofibers, nanofibers with good antibacterial properties were obtained [86,101].

Generally, the advantages of inorganic materials are stable, have long-lasting antibacterial properties, and have good performance in wound dressings, food packaging, water treatment, and other applications. However, due to the diversity of antibacterial mechanisms, the antibacterial properties of inorganic materials are affected by many factors. For example, Raghupathi et al. proved that the antibacterial properties of ZnO are size-dependent [102]. The nanorod-shaped ZnO prepared by Zubair had better antibacterial activity than the hierarchical flower-type ZnO [103]. In the next chapter, the antibacterial mechanism of inorganic materials will be described in detail.

### 3.3. Natural Raw Materials and Extracts

Natural polymers often have excellent biological safety and are widely used in the biomedical field [104,105]. At present, the natural polymer materials used in electrospinning are divided into proteins and polysaccharides. The proteins include gelatin, collagen, silk fibroin, and zein, and the polysaccharides include cellulose acetate, hyaluronic acid, chitosan, and sodium alginate [106,107]. The above materials, except chitosan, have no antibacterial activity, so this chapter will mainly discuss chitosan and its antibacterial material extracts used in electrospinning.

Chitosan is a natural polysaccharide polymer obtained via deacetylation of chitin. It has good biocompatibility, biodegradability, and antibacterial activity. However, due to its polycation property, rigid chemical structure, and intermolecular interaction in the solution, the formation of chain entanglement is limited, which makes it difficult to form nanofibers via electrospinning [108,109]. Electrospinning chitosan with synthetic polymers such as PEO and PVA is the simplest and most effective way to obtain chitosan nanofibers as these polymers can improve the performance of the nanofibers in the process of electrospinning [110,111]. Kegere et al. mixed chitosan and Bidens bipinnata extract (EXT) into a PVA solution [112]. However, during the electrospinning process they found that the nanofibers were broken, beaded, and ribbon-shaped. After adjusting the concentration, flow rate, and voltage, the optimal spinning conditions were successfully found to obtain nanofibers with good morphology and uniform size. In the work of Bhattarai et al., a typical electrospinning method of mixing a chitosan solution with a PEO solution was used, and the nonionic surfactant Triton X-100 and DMSO were added to improve the uniformity of the fiber structure [113]. Hassan et al. mixed chitosan, starch, and a PVA solution to prepare the nanofiber membrane. 

However, there are still some problems when preparing chitosan nanofibers by electrospinning. For example, the solvent of chitosan is generally toxic or an irritating liquid such as acetic acid, trifluoroacetic acid, HFIP, CF, etc.; there are fiber beads in the process of electrospinning chitosan; the mechanical properties of the nanofibers are weak [114,115,116]. To avoid the use of toxic or irritating solvents such as HFIP, CF, TFA, and acetic acid, chitosan can be modified and made water-soluble. The current modification methods involve the preparation of carboxylated chitosan and chitosan salt [117,118,119,120]. This includes carboxymethyl chitosan, carboxyethyl chitosan, quaternized chitosan, etc. [121,122,123]. Among them, carboxymethyl chitosan is one of the most frequently used chitosan derivatives to obtain nanofibers via electrospinning, and quaternized chitosan is another frequently used chitosan derivative that is chosen to form nanofibers because of its excellent antibacterial activity and biocompatibility [124].

In addition to chitosan, some natural plant extracts can also be used as electrospinning antibacterial agents, such as thyme essential oil, moringa leaf extract, clerodendrum phlomidis leaf extract, cleome droserifolia extract, allium sativum extract, and sophora flavescens extract [125,126,127,128,129,130]. All these extracts bring good antibacterial properties to nanofibers and greatly expand the range of spinning materials.

## 4. Antibacterial Mechanisms

Although the antibacterial materials used in the field of electrospinning have good antibacterial properties, their antibacterial emphases are different. In view of the different cell wall structures of gram-negative bacteria and gram-positive bacteria, some antibacterial materials have a better antibacterial effect on gram-negative bacteria, while others have a better antibacterial effect on gram-positive bacteria [131,132]. The classification and listing of the antibacterial mechanisms of these materials will help researchers develop appropriate antibacterial materials according to their different needs.

### 4.1. Synthetic Organics

Quaternary ammonium compounds are widely used cationic surfactants and antibacterial agents. It is generally believed that the antibacterial mechanism of quaternary ammonium salts is that the *n* atoms in the structure exhibit cationic charges and attract the membrane through ionic interactions, thus destroying the structure, resulting in the leakage of intracellular components and cell decomposition [133]. The antibacterial properties of quaternary ammonium compounds with 12–18 carbon chains have been proved, but both the molecular weight and the length of N alkyl chains affect their antibacterial activity [134,135,136]. Lv et al. evaluated the corresponding antibacterial activity of monomers and related polymers by determining the minimum bactericidal concentration (MBC), minimum inhibitory concentration (MIC), and diameter of the inhibition zone of alkyl quaternary ammonium salts with different chain lengths [137]. The results showed that as the length of the alkyl chain increased, the quaternary ammonium monomers enhanced the antibacterial activity, and the polymers exhibited higher bactericidal activity. It has been suggested that the increase of the alkyl chain length contributes to the hydrophobic interaction between the lipid layer and the side chain of the cell wall, and the strong reaction between the alkyl chain and the cytoplasmic membrane of the bacteria is enhanced, thereby enhancing the antibacterial activity.

Chlorhexidine is a hydrophobic and lipophilic molecule with a positive charge. It can penetrate into bacteria through some types of transport and then exchange with phospholipids and lipopolysaccharides of the bacterial membrane [138]. Similar to quaternary ammonium compounds, cationic CHX molecules can be linked to anionic compounds such as free sulfates, lipopolysaccharide phosphate groups, and protein carboxyl groups [139]. In the interaction with bacteria, the interaction between the positively charged chlorhexidine molecule and the negatively charged phosphate group on the bacterial wall will change the cell osmotic balance and eventually lead to membrane leakage [140]. When the concentration is lower than 2%, chlorhexidine will also reduce the permeability of the cell plasma membrane. It changes the ability of bacteria to regulate osmotic pressure and modifies some enzymes, resulting in abnormal levels of potassium and phosphorus ions. On the contrary, CHX has a bactericidal effect at a higher concentration of more than 2%, in which the precipitation of cytoplasmic inclusions will lead to bacterial death [141].

### 4.2. Inorganic Nanoparticles 

#### 4.2.1. Metal

**Figure 8:**
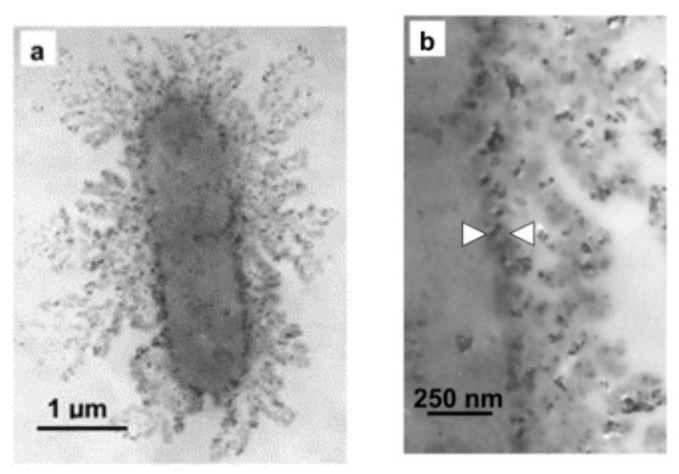
TEM images of *E. coli* cell treated with 50 μg·cm^−3^ of silver nanoparticles in liquid LB medium for 1 h (**a**) and an enlarged view of the membrane of this cell (**b**). Reprinted with permission from ref. [143]. Copyright 2004 Elsevier.

Among many antibacterial metals, silver nanoparticles have broad-spectrum antibacterial properties that have excellent antibacterial properties against bacteria, fungi, viruses, and other microorganisms [142]. For example, Figure 8 shows the incorporation of silver nanoparticles into the membrane structure. The antibacterial mechanism of silver nanoparticles can be attributed to the following three aspects. (1) Silver nanoparticles will accumulate on the cell membrane and attack the phospholipid layer of the cell membrane, resulting in the loss of integrity of the cell membrane and the leakage of intracellular substances. Sondi et al. found that Ag nanoparticles adsorbed on the cell membrane increased the cell permeability and led to the intracellular material flowing outward [143]. Morones et al. indicated that Ag nanoparticles bind to thiol groups of proteins on cell membranes [144]. Rafi et al. suggested that the direct damage of Ag nanoparticles to cell membranes was due to the charge interaction between negatively charged bacteria and positively charged nanoparticles [145]. (2) The release of Ag^+^ is an important part of the antibacterial mechanism of Ag nanoparticles. Ag^+^ can bind to electron donors in biomolecules containing sulfur, oxygen, or nitrogen. It also acts on cell enzymes and proteins, affects cell respiration and ion transmembrane movement, and eventually leads to cell death [146,147]. In an earlier study, cysteine was added to cells exposed to silver nanoparticles. Since cysteine can bind to Ag^+^ and prevent the toxic release of Ag^+^, the antibacterial activity of Ag nanoparticles decreased significantly after adding cysteine, which indicates that the antibacterial activity of silver nanoparticles partly contributes to the release of Ag^+^ [148]. (3) Ag nanoparticles can produce ROS such as hydroxyl radical (·OH) and superoxide anion (O_2_^−^) and induce oxidative stress of bacteria. For example, Danilczuk et al. found free radicals produced by silver nanoparticles via electron spin resonance (ESR) [149]. Similarly, Kim also found that adding antioxidant N-acetylcysteine to the culture medium could counteract the bactericidal effect of Ag nanoparticles, which proved that the antibacterial activity of Ag nanoparticles was related to the free radicals produced [148].

#### 4.2.2. Metal Oxide

At present, the antibacterial mechanism of metal oxides represented by zinc oxide is proposed in three aspects:

(1) More ROS was produced.

For example, Qiu et al. showed that both ZnO and CuO produced a large amount of hydrogen peroxide under UV irradiation, but the metal ion release was basically the same as that in the dark, which proved that the enhancement of the antibacterial effect after UV treatment should be attributed to the production of hydrogen peroxide [150]. Metal oxides such as ZnO and TiO_2_ can produce ROS not only under ultraviolet light but also under dark conditions [151,152,153]. This discovery makes up for the deficiency of ROS antibacterial mechanisms. In addition, Raghupathi et al. used six different sizes of ZnO nanoparticles to explore the effect of size on the antibacterial activity. The results showed that the antibacterial activity of ZnO nanoparticles against *S. aureus* gradually increased with a decrease in size [102]. The transcriptional analysis of some genes directly involved in ROS neutralization showed that the expression of these genes was not enhanced, which suggested that ROS might not be the only factor affecting the antibacterial activity of nano ZnO.

(2) Release of Zn^2+^ and its reaction with cell membranes and cytoplasmic components.

Some scholars believe that the antibacterial properties of metal oxides such as ZnO are related to metal cations. Similar to metal particles, these metal oxides will also release metal cations, damage cell membranes and cell walls, and change membrane permeability [154,155,156,157]. But this point is more controversial. Some scholars believe that the low concentration of Zn^2+^ in ZnO suspension is not the main reason for the antibacterial properties of ZnO [102]. In order to verify the antibacterial effect of ZnO, Zhang et al. prepared a ZnCl_2_ solution with a concentration of 7.3 × 10^−5^ m and found that the solution had no antibacterial effect on bacteria [158]. Considering the size, morphology, and operating conditions of ZnO can affect its antibacterial activity, the controversy may be caused by these factors [159,160,161,162,163].

(3) Electrostatic force leads to the accumulation of ZnO nanoparticles on the surface of bacteria, causing membrane damage and cell function disorder.

Under biological pH values, due to the dissociation of the carboxyl group and other functional groups, the surface of bacterial cells is negatively charged. Since the surface of ZnO is positively charged, this leads to strong electrostatic attraction between them [164]. When it accumulates on the surface of cell membranes, ZnO will change the surface potential of cell membranes, leading to membrane blistering, ruptures, morphological changes, and increased permeability. Eventually, the intracellular fluid and components leak out [165]. This point has been reported in many studies and there is basically no controversy.

#### 4.2.3. Carbon-Based Nanomaterials

The antibacterial properties of carbon-based materials have been reported in many papers. Among them, GO has the highest antibacterial activity in graphene derivatives, and its two-dimensional sheet can be used as the growth anchor of other nanoparticles and composites with a variety of materials [94,166]. Due to its easy functionalization, high dispersibility in aqueous media, and relatively good biocompatibility, GO has the relatively widest application in the field of antimicrobial electrospinning. The antibacterial mechanism of GO can be divided into two parts: (1) damage to the cell membrane; (2) oxidative stress caused by ROS or charge transfer. 

In the early work of Liu et al., the antibacterial activities of graphite, graphite oxide, graphene oxide, and reduced graphene oxide were systematically compared under similar concentrations and incubation conditions using *E. coli* as the model [166]. The results first showed that graphene oxide had the highest antibacterial activity, and the antibacterial mechanism was further summarized into three steps: (1) deposition of graphene on the surface of bacteria; (2) damaging cell membrane and inducing membrane stress through direct contact; (3) oxidative stress independent of superoxide anion. Molecular dynamics simulations based on graphene showed that the nanosheets further contacted with the membrane through van der Waals force and hydrophobic interaction, which destroyed the cell membrane and extracted a large amount of phospholipids from the cell membrane [167]. The antibacterial effect of these physical actions largely depends on the size of the GO sheet. Yi et al., further proved that micron-sized graphene preferentially exhibits a vertical configuration relative to the cell wall, while the nanoscale sheet will adopt a position parallel to the lipid. This is driven by the preferential attraction between the hydrocarbon tail of lipids and the lipophilic flat surface of graphene [168]. However, Chen et al. proposed a different view. They believed that large-sized GO would cover and wrap bacterial cells on the membrane, thereby blocking their active sites on the membrane, while small-sized GO would adhere to the bacterial surface and could not effectively isolate the cells from the environment [169]. Dallavalle et al. also believed that larger nanosheets tended to be arranged on the membrane, but differently, he believed that smaller graphene would preferentially penetrate the phospholipid membrane vertically [170]. In a recent study, Yu et al., also believed that reducing GO size could not only reduce the cell capture effect, but enhance the cutting effect [171]. Lu et al. demonstrated the influence of the cutting effect on antimicrobial activity from another angle. When they changed the GO sheet from a horizontal direction to vertical, the membrane damage effect of GO was enhanced. The antibacterial activity of different sizes of GO is still controversial, but the existence of an influencing factor is beyond doubt [172].

On the other hand, oxidative stress caused by ROS or charge transfer is a common antibacterial mechanism of carbon-based materials. Gurunathan et al. observed that after 4 h of co-cultivation with *E. coli*, the ROS levels in GO and reduced graphene oxide (rGO)-treated groups were 3.8 times and 2.7 times higher than the ROS levels in the control group, respectively [173]. After pre-treatment with N-acetylcysteine and reduced glutathione, the oxidative damage of GO/rGO to bacteria was reduced. Therefore, the authors concluded that ROS is the key mechanism of antibacterial activity of GO and rGO, which was further confirmed via nuclear fragmentation assay. In addition, ESR technology combined with spin capture technology has also confirmed the existence of a ROS hydroxyl group in GO suspension [174].

There are other scholars who believe that the oxidative stress caused by graphene-based materials does not necessarily originate from ROS but may be the result of charge transfer. Mangadlao et al. fixed GO nanosheets on a PET substrate to prevent them from penetrating and wrapping the bacteria [175]. Subsequently, antibacterial properties were still observed, proving the antibacterial effect of GO independent of physical effects. Chong et al. studied the possibility of oxidative stress unrelated to ROS [176]. Results indicated that exposure to simulated sunlight accelerated the electron transfer from antioxidant biomolecules to GO, and as a result the antioxidant system was destroyed. Combined with other data, the author concluded that the oxidative stress caused by GO was mainly via accelerated electron transfer.

### 4.3. Chitosan

Chitosan is a kind of natural material that is prepared by deacetylation of chitin and has good antibacterial effect on many kinds of bacteria. Generally speaking, the antibacterial effect of chitosan is mainly related to the leakage of intracellular substances. Chitosan changes the permeability of cell membranes through the interaction between the positive charge of protonated amino groups and the negative charge of bacterial surfaces, resulting in the leakage of intracellular substances and eventually the death of bacteria [177,178]. There are many factors affecting the antibacterial activity of chitosan, such as molecular weight, degree of deacetylation, pH value, and bacterial species. Among them, the effect of molecular weight on the antibacterial properties of chitosan is still controversial. 

Some scholars believe that the antibacterial properties of chitosan increase with the increase of its molecular weight [179]. Zheng et al., showed that for chitosan with a molecular weight of less than 300 kDa, as the molecular weight increases, the antibacterial effect on *S. aureus* was enhanced, but the antibacterial effect on *E. coli* was weakened [180]. However, what can be defined is that the higher the degree of deacetylation of chitosan is, the stronger the antibacterial effect is [131,181,182]. In addition, most scholars believe that the pH value at which chitosan can exert the best antibacterial activity is below 6 [183,184]. For example, Li et al., showed that the antibacterial activities of chitosan suspensions with pH below 5.0 and pH above 9.0 were derived from the effects of acid and alkali, respectively, and pH 6.0 was most suitable for studying the antibacterial activity of chitosan solutions [185]. This may be due to the fact that chitosan has a more positive charge when pH is below 7. With the increase of pH, the positive charge of chitosan decreases greatly, and the solubility of chitosan becomes worse [184,186]. For gram-negative bacteria and gram-positive bacteria, chitosan exhibits different antibacterial effects. In some studies, chitosan showed higher antibacterial activity against gram-negative bacteria [187,188]. Studies have shown that gram-negative bacteria are more sensitive to chitosan because the surface of gram-negative bacteria has a higher negative charge value [189]. However, in other studies, chitosan showed higher antibacterial activity against gram-positive bacteria [120,190,191]. This is because their cell walls are composed of a thick layer of peptidoglycan and phosphoteichoic acid, which is unique to gram-positive bacteria. The phosphorus atoms in the main chain are negatively charged, so they can interact with cationic antibacterial compounds such as chitosan. That is the reason why gram-positive bacteria are more sensitive than gram-negative bacteria [184].

## 5. Application Fields

The preparation of antibacterial nanofibers via electrospinning is becoming widely used in more and more fields. This is mainly attributed to the fact that, compared with other technologies, electrospun nanofibers have a controllable structure, uniform size, flexible functionalization, and a convenient production process [192]. At present, these antibacterial fibers have good performance in such fields as wound dressing, tissue engineering, food packaging, air purification, and water treatment. Table 2 shows the application of electrospinning antibacterial nanofibers in various fields. 

### 5.1. Wound Dressing

**Figure 9:**
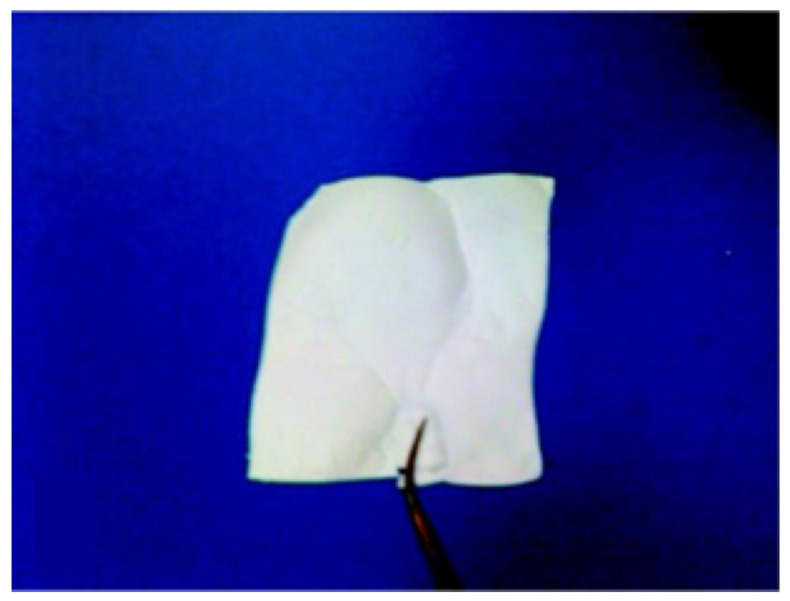
Electrospun PSBMA for non-adherent, superabsorbent, and antimicrobial wound dressing applications. Reprinted with permission from ref. [217]. Copyright 2012 ACS.

When skin or tissue loses its integrity, a suitable wound dressing can protect the wound from bacterial infection and accelerate wound healing. However, traditional wound dressings made of cotton or gauze not only lack necessary antibacterial properties but also need to be changed frequently to keep the wound clean, which may cause secondary injury to the wound. Research indicates that the ideal wound dressing should act as a microbial barrier without affecting gas exchange [179]. The application of electrospinning in the field of wound dressing has the following advantages: (1) the high gas permeability provided by the high porosity of the electrospun fiber; (2) the high absorption caused by the high specific surface area of the fiber; (3) the biocompatibility and antibacterial properties of electrospinning materials. Lalani et al., prepared zwitterionic poly (sulfobetaine methacrylate) (PSBMA) fiber membranes with super water absorption (353% (w/w)) using the electrospinning method (Figure 9) [217]. The excellent water absorption rate was helpful in removing the exudate from the wound in time and keeping the wound moisture. After being immersed in Ag^+^, the fiber membrane exhibited a good bactericidal effect on both gram-negative bacteria and gram-positive bacteria, which could prevent the wound from being infected. In addition, the non-adhesion of the fiber membrane could eliminate the pain of patients when removing the dressing and avoid new wounds after dressing changes. Chen et al. used the dopamine-assisted co-deposition method to prepare bromelain-immobilized PCL nanofibers (BrPDA-PCL). The characterization results showed that the fibers had good mechanical stability, wettability, water vapor transmission rate, biocompatibility, and antibacterial activity [62]. In addition, in vivo experiments showed that the BrPDA-PCL fibers not only increased the wound healing rate but also reduced the related inflammation compared with PCL fibers or the control group. PVA/chitosan/starch nanofibers prepared by Adeli could also be used as a potential wound dressing [218]. Several tests proved that the prepared fibers had the ability to provide a suitable moist environment for the wound, which could make the wound breathe properly, and effectively dealt with the wound exudate. In addition, the fibers exhibited high antimicrobial efficiency against gram-negative *E. coli* and gram-positive *S. aureus* bacteria. 

In addition, in the field of wound dressing and tissue engineering, some specific treatments need polymer scaffolds to provide appropriate drug release profiles. Different manufacturing methods, fiber morphology, and drug loading strongly affect the release curve [219]. However, there are two key points to achieving sustained release: the first is similarity between the polarity of polymer and drug; the second is that the drug must be completely dissolved in the polymer solution. If these requirements are not met, the drugs will be released from the polymer matrix in a short time window [220].

### 5.2. Tissue Engineering

**Figure 10:**
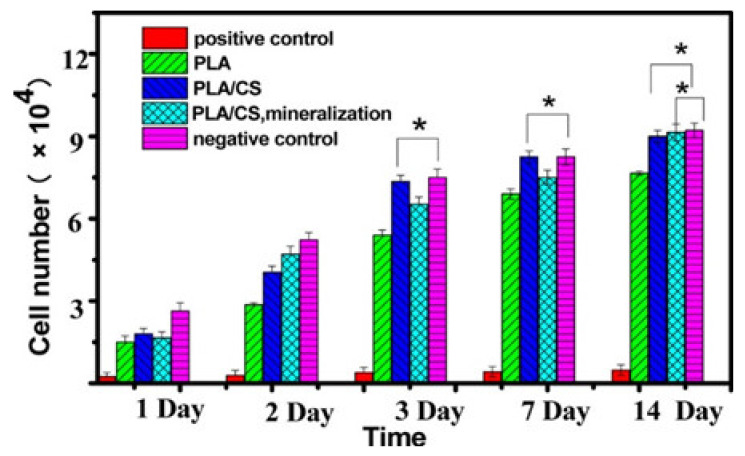
CCK-8 assay of MC3T3-E1 cells in the extract of pure PLA, PLA/CS 70:30, and mineralized PLA/CS scaffolds after culturing for 1, 2, 3, 7, and 14 days (* *p* > 0.05). Reprinted with permission from ref. [225]. Copyright 2017 ACS.

The key to tissue engineering is tissue engineering scaffolds need to have enough functional properties, mechanical strength, and structure to simulate the extracellular matrix to promote cell growth and proliferation [221,222]. Electrospinning has attracted more and more attention due to its unique advantages when applied in tissue engineering. (1) Electrospun nanofibers have the structural characteristics of high porosity, high specific surface area, and nanoscaled fiber size, which can effectively simulate the extracellular matrix and promote cell adhesion, growth, proliferation, and differentiation. (2) The size and morphology of the fibers can be controlled by adjusting the electrospinning parameters. (3) The hydrophilic/hydrophobic properties, antibacterial activity, biocompatibility, and cell proliferation and differentiation can be adjusted by selecting suitable materials [4,223,224]. For example, Xu et al., prepared PLA/CS nanofibers attached with hydroxyapatite (HA) [225]. Through the automatic phase separation between the two incompatible polymers, “island-like” nanoscale bulges were produced on the surface of the fibers. The results showed that the island structures on the fibers were favorable for the proliferation of the cells and had better osteogenic ability (Figure 10). However, the CS/PEO nanofibers doped with cerium bioactive glass prepared by Saatchi eliminated the inherent toxicity of the residual solvents and promoted the adhesion and expansion of cells on the fiber scaffolds. The gelatin/vinyl acetate and PCL scaffolds prepared by Thottapellil et al. could meet the various properties of vascular grafts, such as mechanical properties, degradability, promoting of cell adhesion, proliferation, and differentiation [226]. The characteristics of the scaffolds and non-synthetic nature of smooth muscle cells formed the basis of the generation of grafts based on the ‘outside-in strategy’, which was helpful to improve the endothelialization and patency.

Although there are various advantages and many biocompatibility experiments have been performed, only a few clinical trials have been reported in the literature, and regulatory agencies such as the FDA and EMA have not approved any devices [227]. The reason may be that the toxic solvents used in the electrospinning process probably have a small number of residues and release together with the drugs. Therefore, the use of more green and biocompatible solvents is a promising direction in the field of tissue engineering. Although melt electrospinning can produce nanofibers without solvents, it is difficult to protect drugs from heat and degradation.

### 5.3. Food Packaging

Currently, the food packaging industry relies heavily on the use of non-degradable polymers such as polyethylene (PE), polypropylene (PP), polystyrene (PS), and polyethylene terephthalate (PET) [228]. Although these materials have good mechanical strength and thermal stability and provide high-quality protection for food, their large-scale use and non-biodegradability pose significant environmental risks. The application of electrospinning in the field of food packaging is a relatively novel application method, and the research focus has mainly been on improving biodegradability and antibacterial and antioxidation properties of food packaging [203,206,229]. For example, Aytac et al. electrospun antibacterial zein nanofibers using a non-toxic organic solvent and a mixed antibacterial agent of thyme oil, citric acid, and nisin [202]. Due to the high surface area (21.91 m^2^/g), the antibacterial agent can be released from the fiber to various food simulants within 2 h. Furthermore, the antimicrobial fibers effectively reduced *E. coli* and *L. innocua* populations by ~5 logs after 24 h and 1 h of exposure, respectively, demonstrating the potential of antibacterial food packaging materials. Díez-Pascual et al. prepared chitosan/poly (butylene terephthalate) (CS/PBAT) nanofiber membranes via electrospinning and impregnation [230]. The results showed that there is a strong hydrogen bond interaction between PBAT and CS nanofibers. As a nucleating agent, CS nanofibers improved the crystallization temperature, crystallinity, and thermal stability of co-polyesters. The composite nanofibers have antibacterial activity against common food-borne pathogens *S. aureus*, *B. subtilis*, *S. enteritidis*, and *E. coli.* In addition to the antibacterial effect, CS also has the effect of fruit preservation. During the study, when the CS content was 7 wt%, the composite nanofibers showed the greatest degree of slowing down the metabolism of strawberries and prolonging their shelf life [231]. At the same time, the antibacterial activity of the composite fibers to four kinds of microorganisms reached their peak. 

### 5.4. Water Purification and Air Purification 

The porous structure and high specific surface area of electrospun nanofiber membranes causes them to have unique advantages in the field of membrane filtration, which can reduce pollutants in fluids with low energy and cost. Compared with conventional fiber filters, nanofibers fabricated via electrospinning have an average size that is 800 times smaller. Due to inertial collision and interception, smaller fibers usually provide better filtration efficiency, which makes up for the increased pressure drop compared with larger fibers [232].

As we spend most of our time in an indoor environment, air filtration is an important safeguard to ensure a safe and clean environment. However, when we are outdoors or under specific working conditions, effective personal protective equipment can also filter and absorb dust as well as toxic and harmful substances in the air to keep us safe [192,233]. Many nanofibers prepared via electrospinning have been successfully used to produce high-performance air filters, proving that electrospun fibers are an ideal choice for the production of filters with high filtration efficiency and low resistance [234,235]. Zhang et al., developed a highly efficient PI nanofiber air filter through electrospinning that had a long service life and could keep the removal efficiency (99.5%) of PM2.5 unchanged in the range 25–370 °C [236]. Generally, compared with the common commercial air filters, this filter had higher filtration efficiency, lower pressure drops, and better temperature stability. In addition, PAN nanofibers doped with attapulgite also had good air filtration performance (high air filtration efficiency and relatively low pressure) [237]. The CS/PVA nanofibers doped with SiO_2_/Ag nanoparticles prepared by Zhu et al. not only had good air filtration performance but also showed good antibacterial activity and biocompatibility, which demonstrated excellent prospects in the field of personal protective masks [216].

Polluted water can disturb the ecosystem and pose a serious threat to humans and animals. With an increase in human activities, how to deal with pollutants in water in a low-cost and efficient way has become a hot topic. Magnetic nanomaterials have attracted more and more attention in wastewater treatment due to their easy recovery via external magnets, while iron (Fe) is widely used due to its remarkable magnetism. The polymer can avoid the oxidation and flocculation of metal nanoparticles, thus supporting the excellent stability of these nanoparticles in the composite [238]. By electrospinning polymer-based magnetic materials such as Fe, we can not only provide effective support and fixation for these magnetic materials, but also make use of the large contact area and appropriate porosity of electrospun fiber membranes to play the role of membrane filtration so as to obtain composite nanofiber membranes with appropriate osmotic pressure, good adsorption capacity, and filtration efficiency. If hollow-structure nanofibers were combined with Fe (OH)_3_ nanoparticles, nanofiber membranes with good mechanical strength and a water flux of 11,200 L/m^2^/h/bar could be obtained. In adsorption experiments of phosphate, Cr (VI), and Congo red, adsorption effects of 172.41 mg/g, 63.29 mg/g, and 735.29 mg/g were shown, respectively [239]. The use of magnetic particles is often limited due to their easy oxidation/dissolution [240]. In order to protect Fe_2_O_3_ magnetic particles from oxidation, core–shell structures were prepared via coaxial electrospinning γ- Fe_2_O_3_@Ti_0.9_Si_0.1_O_2_. Nanofibers and Ti_0.9_Si _0.1_O_2_ in the shell can not only protect the magnetic Fe_2_O_3_ in the core from oxidation, but also can be used as a visible light photocatalyst to further degrade dyes in wastewater [241]. Kim et al. prepared a chlorinated meta-aramid membrane for water disinfection via electrospinning. Its advantage was that the fiber membrane had sufficient antibacterial effect against *E. coli* and *S. aureus*, and the chlorine lost after filtration could be supplemented by chlorination, revealing the innovative application of meta-aramid membranes in the water industry [242]. Lignin also has a good adsorption effect on MB [209]. Compared with commercial granular activated carbon, the adsorption capacity of lignin nanofiber membranes is about 10 times that of traditional activated carbon, the adsorption kinetics 2 times, and the permeability is increased by 6 times. This is mainly due to the high specific surface area (583 cmg/m^2^), large average pore size (258 nm/m^2^), and high porosity (583 nm/m^2^). When modeled in a hypothetical water purification process, the membrane could reduce energy consumption by 87%, saving $300,000 in pumping costs and $40,000 in regeneration costs per year.

## 6. Conclusions and Prospects

In this review, the morphology, materials, antibacterial mechanisms, and applications of electrospun nanofibers were summarized. 

(1) The characteristics of different morphologies of nanofibers was suggested. For example, porous nanofibers have a larger specific surface area, which is more conducive to the play of antibacterial activity. For nanofibers with a core–shell structure, the antibacterial agent can be wrapped by the shell, which is more conducive to the slow release of antibacterial activity.

(2) The antibacterial materials electrospun in antibacterial nanofibers were divided into synthetic organics, inorganic materials, and natural antibacterial materials, and some of the representations were introduced.

(3) The antibacterial mechanisms of these antibacterial materials and the factors influencing their antibacterial activity were discussed.

(4) The applications of these antibacterial nanofibers were summarized, and the unique application advantages of these antibacterial nanofibers prepared via electrospinning were described.

It can be seen that electrospun antibacterial nanofibers have many attractive advantages and prospects in different fields. However, the industrialization of electrospinning is still far from meeting large-scale application needs, and the industrialization of some nanofibers with special morphologies is more difficult. Therefore, more research on the mechanisms and technology is needed.

## Figures and Tables

**Table 1:** Applications of organic antibacterial agents in electrospinning.

Polymer	Active Agent	Antibacterial Effect	Reference
PVA	Chitosan/tetracycline hydrochloride	The antibacterial diameter of *E. coli* was 8.8 ± 0.4 mm The diameter of *S. epidermidis* was 15.6 ± 0.3 mm The diameter of S. aureus was 19.6 ± mm	[69]
Thermoplastic polyurethane (TPU)	Tetracycline hydrochloride/montmorillonite	The diameter of *S. aureus* was 37 mm. The bacteriostatic diameter of *E*. *coli* was 34 mm	[70]
PLGA/gum tragacanth	Tetracycline hydrochloride	*S. aureus* and *P. aeruginosa* were used as a model for the inhibition zone experiment	[71]
PEO/Chitosan	Chlorhexidine/silver nanoparticles	*S. aureus* were used as a model for the inhibition zone experiment	[72]
PHB/PEO	Chlorhexidine	Minimum inhibitory concentration: *E. coli* 2–8 μg·mL^−1^ *S. aureus* 0.5–4 μg·mL^−1^	[73]
Nylon/poly(bisphenol A carbonate)	Cetyltrimethyl ammonium bromide	The average logarithmic attenuation of *S. aureus* was 3.3 and 2 when the mass fraction was 5% and 10%, respectively	[74]
PVA	Quaternary ammonium salts	It had 99.9% antibacterial activity against *E. coli* and *S. aureus*	[75]
PCL	Quaternary ammonium salts	The antibacterial activity against *E. coli* was 99.85% ± 0.26 and 99.74% ± 0.44, respectively	[76]

**Table 2:** Application of electrospinning antibacterial nanofibers in various fields.

Polymer	Additives	Application Field	Reference
PCL	Quercetin/GO	Wound dressing	[193]
PCL	Bromelain/PDA	Wound dressing	[62]
PVP/ethyl cellulose (EC)	Ciprofloxacin/Ag nanoparticles	Wound dressing	[194]
PVA	CS/copper-based MOF	Wound dressing	[195]
PCL	Quaternary ammonium salt	Wound dressing	[76]
Hydrophilic amino modified zwitterionic poly (sulfobetaine methacrylate)	Halloysite nanotubes loaded with tetracycline hydrochloride (TCH)	Wound dressing	[196]
Poly (hydroxybutyrate)/poly (epsilon caprolactone)/sol-gel silica (PHB/PCL/SGS)	Levofloxacin (LFX)	Tissue engineering	[197]
Chitosan/alginate	Gentamicin	Tissue engineering	[198]
PLA/gelatin	Ag nanoparticles	Tissue engineering	[199]
PVA	Nano demineralized bone matrix/carbon nanotubes	Tissue engineering	[200]
CS/PCL	Halloysite nanotubes loaded with chlorogenic acid	Tissue engineering	[201]
Zein	Thyme oil/citric acid/nisin	Food packaging	[202]
Gelatin	Peppermint essential oil/chamomile essential oil	Food packaging	[203]
PVA	Pomegranate peel extract/sodium dehydroacetate	Food packaging	[204]
Zein/PLA	Carvacrol	Food packaging	[205]
PVA	Ag nanoparticles	Food packaging	[206]
Polyacrylonitrile (PAN)	ZnO/CS	Water purification	[207]
Polyvinylidene fluoride	Tetrafluoromethane plasma	Water purification	[208]
PAN	Lignin	Water purification	[209]
β-cyclodextrin/cellulose (β-CD/CA)	Ag/Fe	Water purification	[210]
PLA	Zeolite imidazole framework/graphene oxide	Water purification	[211]
polyacrylonitrile	Palladium acetylacetonate/multi-walled carbon nanotubes	Air purification	[212]
Polyvinyl alcohol/cellulose nanocrystals		Air purification	[213]
PVA	Sodium lignosulfonate	Air purification	[214]
Polyvinyl alcohol/polyacrylic acid	Silica/silver nanoparticles	Air purification	[215]
CS/PVA	SiO_2_/Ag nanoparticles	Air purification	[216]
